# 2-Amino-4,6-dimethyl­pyrimidinium dihydrogenphosphate

**DOI:** 10.1107/S160053680903880X

**Published:** 2009-10-03

**Authors:** Cui-Hua Lin, Nai-Sheng Liu, Fang-Fang Jian

**Affiliations:** a Microscale Science Institute, Department of Chemistry and Chemical Engineering, Weifang University, Weifang 261061, People’s Republic of China; b Journal Editorial Department, Weifang University, Weifang 261061, People’s Republic of China; c Microscale Science Institute, Weifang University, Weifang 261061, People’s Republic of China

## Abstract

In the crystal structure of the title compound, C_6_H_10_N_3_^+^·H_2_PO_4_^−^, the cations and anions are linked by inter­molecular O—H⋯O and N—H⋯O hydrogen bonds, forming a two-dimensional network. Additional stabilization is provided by weak inter­molecular C—H⋯O inter­actions. N—H⋯N inter­actions are also present.

## Related literature

Five and six-membered heterocyclic compounds often exist in biologically active natural products and synthetic compounds of medicinal inter­est, see: Gilchrist (1998 ▶). For methyl­pyrimidines as precursors to potentially bioactive pyrimidine derivatives, see: Xue *et al.* (1993 ▶). For Ru complexes of pyrim­idine with an –NH_2_ substituent, see: Zhu *et al.* (2008 ▶). For bond-length data, see: Allen *et al.* (1987 ▶).
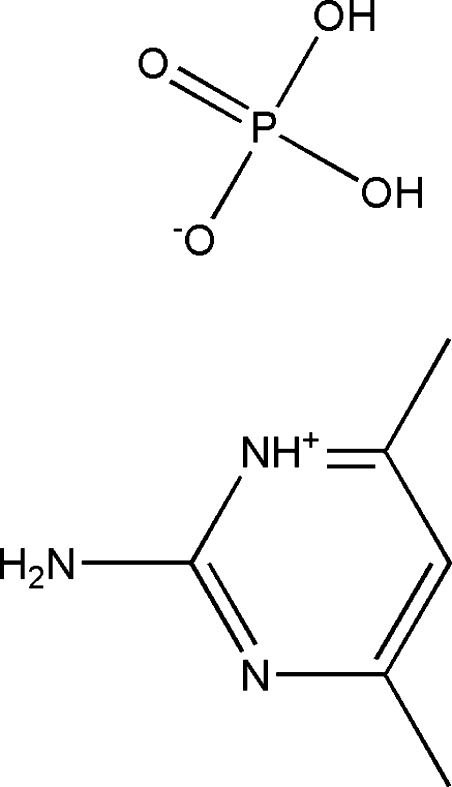


## Experimental

### 

#### Crystal data

C_6_H_10_N_3_^+^·H_2_PO_4_^−^
*M* *_r_* = 221.16
Monoclinic, 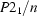

*a* = 11.743 (2) Å
*b* = 4.8266 (10) Å
*c* = 16.940 (3) Å
β = 95.55 (3)°
*V* = 955.6 (3) Å^3^
*Z* = 4
Mo *K*α radiation
μ = 0.28 mm^−1^
*T* = 293 K
0.20 × 0.15 × 0.11 mm

#### Data collection

Bruker SMART CCD diffractometer
Absorption correction: none
8743 measured reflections
2193 independent reflections
1973 reflections with *I* > 2σ(*I*)
*R*_int_ = 0.021

#### Refinement

*R*[*F* ^2^ > 2σ(*F* ^2^)] = 0.035
*wR*(*F* ^2^) = 0.108
*S* = 1.08
2193 reflections
133 parameters
H-atom parameters constrained
Δρ_max_ = 0.40 e Å^−3^
Δρ_min_ = −0.41 e Å^−3^

### 

Data collection: *SMART* (Bruker, 1997 ▶); cell refinement: *SAINT* (Bruker, 1997 ▶); data reduction: *SAINT*; program(s) used to solve structure: *SHELXS97* (Sheldrick, 2008 ▶); program(s) used to refine structure: *SHELXL97* (Sheldrick, 2008); molecular graphics: *PLATON* (Spek, 2009 ▶); software used to prepare material for publication: *SHELXTL* (Sheldrick, 2008).

## Supplementary Material

Crystal structure: contains datablocks I, global. DOI: 10.1107/S160053680903880X/lh2909sup1.cif

Structure factors: contains datablocks I. DOI: 10.1107/S160053680903880X/lh2909Isup2.hkl

Additional supplementary materials:  crystallographic information; 3D view; checkCIF report

## Figures and Tables

**Table 1:** Hydrogen-bond geometry (Å, °)

*D*—H⋯*A*	*D*—H	H⋯*A*	*D*⋯*A*	*D*—H⋯*A*
N1—H1*A*⋯O1	0.93	1.75	2.667 (2)	168
O2—H2*A*⋯O3^i^	0.87	1.70	2.560 (2)	171
N3—H3*A*⋯O3	0.82	2.02	2.831 (2)	170
N3—H3*B*⋯N2^ii^	0.93	2.07	3.004 (2)	178
O4—H4*A*⋯O1^iii^	0.92	1.68	2.594 (2)	171
C3—H3*D*⋯O2^iv^	0.93	2.40	3.319 (2)	171

Symmetry codes: (i) 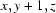; (ii) 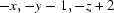; (iii) 
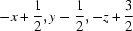
; (iv) 
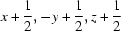
.

**Fig. 1.**
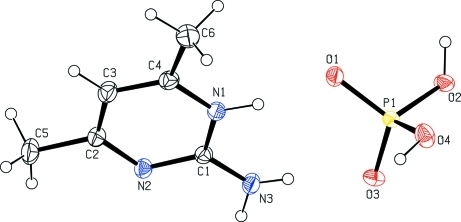
The molecular structure of the title compound showing 30% probability displacement ellipsoids and the atom-numbering scheme.

## supplementary crystallographic information

### Comment

Five and six-membered heterocyclic compounds are important constituents that often exist in biologically active natural products and synthetic compounds of medicinal interest (Gilchrist, 1998). As useful precursors to potentially bioactive pyrimidine derivatives, methylpyrimidine has attracted considerable attention for many years (Xue *et al.*, 1993). In recent years, Ru complexes of pyrimidine with an –NH2 substituent have been synthesized (Zhu *et al.*, 2008). The title compound(I), was synthesized and we report herein its crystal structure.

The molecular structure of the title compound is shown in Fig. 1. There is one 2-Amino-4,6-dimethylpyrimidine cation and one dihydrogenphosphate anion in the asymmetric unit. All bond lengths are within the normal ranges (Allen *et al.*,  1987). In the crystal structure, cations and anions are linked by intermolecular O-H···O and N-H···O hydrogen bonds to form a two-dimensional network. Additional stabilization is provided by weak intermolecular C—H···O interactions.

### Experimental

A mixture of guanidine hydrochloride (0.1 mol), acetyl acetone (0.2 mol), sodium carbonate (0.03 mol) and phosphoric acid (0.1 mol) was stirred with water (30 mL) for 5 h to afford the title compound (yield 78%). Single crystals suitable for X-ray measurements were obtained by recrystallization of the title compound from water at room temperature.

### Refinement

H atoms bonded to C atoms were fixed geometrically and and included in a riding-model approximation with C—H = 0.93-0.96 Å and *U*_iso_(H)=1.2–1.5*U*_eq_(C). H atoms bonded to N and O atoms were incuded in their 'as found' locations with refined isotropic displacement parameters,

### Crystal data

**Table:**

C_6_H_10_N_3_^+^·H_2_PO_4_^−^	*F*(000) = 464
*M**_r_* = 221.16	*D*_x_ = 1.537 Mg m^−^^3^
Monoclinic, *P*2_1_/*n*	Mo *K*α radiation, λ = 0.71073 Å
Hall symbol: -P 2yn	Cell parameters from 1973 reflections
*a* = 11.743 (2) Å	θ = 3.5–27.5°
*b* = 4.8266 (10) Å	µ = 0.28 mm^−^^1^
*c* = 16.940 (3) Å	*T* = 293 K
β = 95.55 (3)°	Block, colorless
*V* = 955.6 (3) Å^3^	0.20 × 0.15 × 0.11 mm
*Z* = 4	

### Data collection

**Table:**

Bruker SMART CCD diffractometer	1973 reflections with *I* > 2σ(*I*)
Radiation source: fine-focus sealed tube	*R*_int_ = 0.021
graphite	θ_max_ = 27.5°, θ_min_ = 3.5°
φ and ω scans	*h* = −15→15
8743 measured reflections	*k* = −6→5
2193 independent reflections	*l* = −21→21

### Refinement

**Table:**

Refinement on *F*^2^	Primary atom site location: structure-invariant direct methods
Least-squares matrix: full	Secondary atom site location: difference Fourier map
*R*[*F*^2^ > 2σ(*F*^2^)] = 0.035	Hydrogen site location: inferred from neighbouring sites
*wR*(*F*^2^) = 0.108	H-atom parameters constrained
*S* = 1.08	*w* = 1/[σ^2^(*F*_o_^2^) + (0.0686*P*)^2^ + 0.3094*P*] where *P* = (*F*_o_^2^ + 2*F*_c_^2^)/3
2193 reflections	(Δ/σ)_max_ = 0.001
133 parameters	Δρ_max_ = 0.40 e Å^−^^3^
0 restraints	Δρ_min_ = −0.41 e Å^−^^3^

### Special details

**Table:**

Geometry. All e.s.d.'s (except the e.s.d. in the dihedral angle between two l.s. planes) are estimated using the full covariance matrix. The cell e.s.d.'s are taken into account individually in the estimation of e.s.d.'s in distances, angles and torsion angles; correlations between e.s.d.'s in cell parameters are only used when they are defined by crystal symmetry. An approximate (isotropic) treatment of cell e.s.d.'s is used for estimating e.s.d.'s involving l.s. planes.
Refinement. Refinement of *F*^2^ against ALL reflections. The weighted *R*-factor *wR* and goodness of fit *S* are based on *F*^2^, conventional *R*-factors *R* are based on *F*, with *F* set to zero for negative *F*^2^. The threshold expression of *F*^2^ > σ(*F*^2^) is used only for calculating *R*-factors(gt) *etc*. and is not relevant to the choice of reflections for refinement. *R*-factors based on *F*^2^ are statistically about twice as large as those based on *F*, and *R*- factors based on ALL data will be even larger.

### Fractional atomic coordinates and isotropic or equivalent isotropic displacement parameters (Å^2^)

**Table:**

	*x*	*y*	*z*	*U*_iso_*/*U*_eq_	
P1	0.09666 (3)	0.22149 (8)	0.74781 (2)	0.02327 (14)	
O1	0.17203 (9)	0.3129 (2)	0.82063 (6)	0.0302 (3)	
O2	0.01084 (9)	0.4557 (2)	0.71860 (7)	0.0337 (3)	
H2A	0.0243	0.6265	0.7326	0.085 (9)*	
O3	0.02931 (10)	−0.0362 (2)	0.76133 (7)	0.0339 (3)	
N1	0.20023 (11)	0.0034 (3)	0.95145 (7)	0.0270 (3)	
H1A	0.1936	0.0900	0.9021	0.052 (6)*	
O4	0.16964 (11)	0.1887 (3)	0.67571 (7)	0.0381 (3)	
H4A	0.2241	0.0508	0.6822	0.084 (9)*	
N3	0.02982 (11)	−0.2279 (3)	0.91928 (8)	0.0322 (3)	
H3B	−0.0198	−0.3692	0.9308	0.032 (4)*	
H3A	0.0243	−0.1573	0.8748	0.044 (6)*	
C1	0.12099 (12)	−0.1846 (3)	0.96966 (8)	0.0252 (3)	
N2	0.13194 (11)	−0.3247 (3)	1.03924 (7)	0.0282 (3)	
C4	0.29458 (13)	0.0550 (3)	1.00208 (9)	0.0300 (3)	
C2	0.22244 (14)	−0.2691 (3)	1.09017 (9)	0.0294 (3)	
C3	0.30667 (13)	−0.0793 (4)	1.07340 (9)	0.0341 (3)	
H3D	0.3695	−0.0453	1.1099	0.041*	
C6	0.37991 (16)	0.2522 (4)	0.97449 (12)	0.0417 (4)	
H6A	0.3427	0.4240	0.9594	0.063*	
H6B	0.4128	0.1748	0.9297	0.063*	
H6C	0.4391	0.2851	1.0166	0.063*	
C5	0.23093 (16)	−0.4213 (4)	1.16774 (9)	0.0429 (4)	
H5A	0.1664	−0.5429	1.1690	0.064*	
H5B	0.2315	−0.2905	1.2105	0.064*	
H5C	0.3002	−0.5280	1.1734	0.064*	

### Atomic displacement parameters (Å^2^)

**Table:**

	*U*^11^	*U*^22^	*U*^33^	*U*^12^	*U*^13^	*U*^23^
P1	0.0252 (2)	0.0188 (2)	0.0253 (2)	0.00191 (12)	−0.00052 (14)	0.00042 (12)
O1	0.0312 (6)	0.0308 (6)	0.0274 (5)	−0.0050 (4)	−0.0039 (4)	0.0044 (4)
O2	0.0333 (6)	0.0214 (5)	0.0437 (6)	0.0054 (4)	−0.0103 (4)	−0.0035 (4)
O3	0.0404 (6)	0.0207 (5)	0.0400 (6)	−0.0048 (4)	0.0005 (5)	0.0003 (4)
N1	0.0281 (6)	0.0277 (6)	0.0250 (6)	−0.0035 (5)	0.0012 (4)	0.0012 (5)
O4	0.0470 (7)	0.0380 (7)	0.0305 (6)	0.0135 (5)	0.0099 (5)	0.0051 (5)
N3	0.0327 (7)	0.0363 (7)	0.0263 (7)	−0.0087 (5)	−0.0031 (5)	0.0042 (5)
C1	0.0265 (7)	0.0256 (7)	0.0236 (7)	−0.0009 (5)	0.0026 (5)	−0.0008 (5)
N2	0.0303 (6)	0.0310 (7)	0.0233 (6)	−0.0038 (5)	0.0020 (5)	0.0020 (5)
C4	0.0277 (7)	0.0296 (7)	0.0322 (7)	−0.0034 (6)	0.0008 (5)	−0.0019 (6)
C2	0.0327 (8)	0.0313 (8)	0.0239 (7)	0.0003 (6)	0.0011 (5)	−0.0007 (5)
C3	0.0310 (8)	0.0391 (9)	0.0308 (7)	−0.0049 (7)	−0.0040 (6)	0.0005 (6)
C6	0.0346 (9)	0.0423 (10)	0.0475 (10)	−0.0125 (7)	−0.0002 (7)	0.0068 (7)
C5	0.0479 (10)	0.0532 (11)	0.0261 (7)	−0.0066 (8)	−0.0032 (6)	0.0089 (7)

### Geometric parameters (Å, °)

**Table:**

P1—O3	1.5033 (12)	N2—C2	1.330 (2)
P1—O1	1.5128 (12)	C4—C3	1.366 (2)
P1—O2	1.5626 (11)	C4—C6	1.490 (2)
P1—O4	1.5665 (12)	C2—C3	1.397 (2)
O2—H2A	0.8679	C2—C5	1.500 (2)
N1—C1	1.3566 (19)	C3—H3D	0.9300
N1—C4	1.3568 (19)	C6—H6A	0.9600
N1—H1A	0.9317	C6—H6B	0.9600
O4—H4A	0.9225	C6—H6C	0.9600
N3—C1	1.3195 (19)	C5—H5A	0.9600
N3—H3B	0.9302	C5—H5B	0.9600
N3—H3A	0.8243	C5—H5C	0.9600
C1—N2	1.3542 (19)		
			
O3—P1—O1	113.08 (6)	C3—C4—C6	124.41 (15)
O3—P1—O2	108.30 (7)	N2—C2—C3	122.50 (14)
O1—P1—O2	110.84 (7)	N2—C2—C5	116.78 (14)
O3—P1—O4	111.77 (7)	C3—C2—C5	120.72 (15)
O1—P1—O4	110.13 (7)	C4—C3—C2	118.42 (14)
O2—P1—O4	102.18 (7)	C4—C3—H3D	120.8
P1—O2—H2A	120.4	C2—C3—H3D	120.8
C1—N1—C4	121.00 (13)	C4—C6—H6A	109.5
C1—N1—H1A	120.3	C4—C6—H6B	109.5
C4—N1—H1A	118.5	H6A—C6—H6B	109.5
P1—O4—H4A	113.9	C4—C6—H6C	109.5
C1—N3—H3B	117.8	H6A—C6—H6C	109.5
C1—N3—H3A	120.9	H6B—C6—H6C	109.5
H3B—N3—H3A	119.9	C2—C5—H5A	109.5
N3—C1—N2	119.12 (14)	C2—C5—H5B	109.5
N3—C1—N1	119.36 (13)	H5A—C5—H5B	109.5
N2—C1—N1	121.51 (13)	C2—C5—H5C	109.5
C2—N2—C1	117.81 (13)	H5A—C5—H5C	109.5
N1—C4—C3	118.72 (14)	H5B—C5—H5C	109.5
N1—C4—C6	116.85 (14)		

### Hydrogen-bond geometry (Å, °)

**Table:**

*D*—H···*A*	*D*—H	H···*A*	*D*···*A*	*D*—H···*A*
N1—H1A···O1	0.93	1.75	2.667 (2)	168
O2—H2A···O3^i^	0.87	1.70	2.560 (2)	171
N3—H3A···O3	0.82	2.02	2.831 (2)	170
N3—H3B···N2^ii^	0.93	2.07	3.004 (2)	178
O4—H4A···O1^iii^	0.92	1.68	2.594 (2)	171
C3—H3D···O2^iv^	0.93	2.40	3.319 (2)	171

Symmetry codes: (i) *x*, *y*+1, *z*; (ii) −*x*, −*y*−1, −*z*+2; (iii) −*x*+1/2, *y*−1/2, −*z*+3/2; (iv) *x*+1/2, −*y*+1/2, *z*+1/2.
